# Occurrence and clinical correlates of depressive symptoms in adults with epilepsy: A study in Georgia

**DOI:** 10.1002/epi4.70291

**Published:** 2026-06-08

**Authors:** Ketevan Silagadze, Eka Burkadze, Maka Areshidze, Teimuraz Silagadze, Sofia Kasradze, Josemir W. Sander

**Affiliations:** ^1^ Tbilisi State Medical University (TSMU) Tbilisi Georgia; ^2^ Institute of Neurology and Neuropsychology (INN) Tbilisi Georgia; ^3^ Mental Health Clinic ‘Mental Hub’ Tbilisi Georgia; ^4^ Caucasus International University (CIU) Tbilisi Georgia; ^5^ UCL Queen Square Institute of Neurology London UK; ^6^ Department of Neurology West China Hospital, Sichuan University Chengdu Sichuan China

**Keywords:** Antiseizure medication, psychiatric comorbidity, screening tests, suicidal ideation

## Abstract

**Objective:**

To evaluate the occurrence, clinical correlates, and screening accuracy of depressive symptoms (DS) among adults with epilepsy in Georgia, a country with limited mental health resources.

**Methods:**

We conducted a cross‐sectional study in adults with epilepsy attending a tertiary care center. We assessed DS using two validated tools: the Beck Depression Inventory‐II (BDI‐II) and the Neurological Disorders Depression Inventory for Epilepsy (NDDI‐E), combined with comprehensive clinical and demographic data.

**Results:**

Two hundred and seven adults were included, and DS were identified in 47% using the BDI‐II and 22% using the NDDI‐E, with moderate agreement between the scales (Cohen's κ = 0.469). DS were associated with later epilepsy onset (median 15.0 vs. 13.5 years), frontal lobe epilepsy (26.3% vs. 8.3%), polytherapy with older ASMs (60.7% vs. 42.3%), and ongoing seizures.

**Significance:**

DS represent a substantial psychiatric burden in adults with epilepsy in Georgia, influenced by epilepsy‐related factors and limitations of screening tools. These findings underscore the need for integrated mental health services and routine DS screening in epilepsy care. Longitudinal studies are warranted to clarify causal relationships and inform culturally tailored interventions.

**Plain Language Summary:**

Many people with epilepsy have low mood, but this is often missed and untreated. A recent study in Georgia found that nearly half of people with epilepsy have signs of very low mood. Those who were having regular seizures or taking more medication were more likely to struggle with low mood. In contrast, people who do not have seizures or whose seizures started on the brain's right side showed fewer low mood signs. These findings show that checking mood is just as important as treating their seizures. Routine mental health checks should be part of epilepsy consultations.

**Key points**

- Adults with epilepsy were assessed for depressive symptoms using BDI‐II and NDDI‐E.
- Depressive symptoms affected almost half of them.
- Seizure freedom and right‐sided seizure focus were associated with lower odds of depression.
- Higher ASM burden and polytherapy were more frequent among people with depressive symptoms.
- Most participants with depressive symptoms had underassessed or untreated psychiatric comorbidities.

## 1 INTRODUCTION

Epilepsy is a chronic neurological disorder characterized by recurrent seizures and associated neurobiological, cognitive, psychological, and social consequences, affecting over 50 million people worldwide.^1^ Depression is the most common comorbidity, with prevalence in adults ranging from 20% to over 50%, substantially exceeding rates in the general population.^2^, ^3^, ^4^, ^5^

The relationship between epilepsy and depression is complex and likely bidirectional.^6^, ^7^, ^8^ Psychosocial stressors such as stigma, social isolation,^9^ and chronic uncertainty, along with the effects of antiseizure medications (ASMs), further contribute to the psychiatric burden in this population.^9^, ^10^, ^11^

Comorbid depression in people with epilepsy is associated with adverse outcomes, including poorer seizure control, reduced treatment adherence, reduced quality of life, increased suicidal ideation, higher risk of premature mortality, and greater healthcare use.^12^, ^13^, ^14^, ^15^, ^16^ Despite its clinical significance, depression often remains underdiagnosed and inadequately treated in epilepsy care settings, highlighting the urgent need for systematic screening and integrated treatment approaches.^4^, ^16^

Validated, condition‐specific tools such as the Neurological Disorders Depression Inventory for Epilepsy (NDDI‐E) and the Beck Depression Inventory‐II (BDI‐II) help identify individuals at risk of depression. However, overlapping symptoms like fatigue and anhedonia can complicate detection.^17^, ^18^, ^19^

Understanding psychiatric comorbidities within local healthcare and sociocultural contexts is essential for developing effective, context‐sensitive interventions. However, data on the distribution, severity, and clinical correlates of depressive symptoms (DS) among adults with epilepsy remain limited in many settings, including Georgia.

To address these gaps, we aimed to characterize DS and suicidal ideation in adults with epilepsy attending a tertiary care center, the Institute of Neurology and Neuropsychology (INN). We also evaluated the clinical utility and agreement of two validated depression screening instruments (the BDI‐II and the Georgian NDDI‐E). Lastly, we examined how DS and suicidal ideation relate to clinical factors, including epilepsy type, seizure control, and ASM use.

## 2 METHODS

### 2.1 Study participants

We conducted a prospective single‐center observational study at the Tertiary Epilepsy Center in Tbilisi, Georgia. Participants were enrolled between January 2019 and December 2019. During this period, eligible individuals attending the clinic were invited to participate. This time‐bound, consecutive sampling approach ensured a consistent and structured sampling process.

Eligibility criteria included adults (≥ 18 years) who had been using ASMs for at least 6 months before the study and whose diagnosis of epilepsy was confirmed by board‐certified neurologists according to the classifications of the International League Against Epilepsy (ILAE).^20^, ^21^ Participants were required to have adequate proficiency in Georgian, English, or Russian to complete the structured assessments and to provide informed consent.

Based on medical record review and clinical evaluation, people with documented intellectual disability or progressive cognitive disorders, such as dementia, were excluded.^22^, ^23^ Those with mild cognitive impairment related to epilepsy or its treatment were not excluded. For each participant, 65 variables were collected, encompassing demographic characteristics, clinical epilepsy features, and psychiatric assessment outcomes.

### 2.2 Sample size

This cross‐sectional study potentially targeted an estimated population of approximately 15 600 adults (≥ 18 years) living with epilepsy in Georgia.^24^ Given that a substantial proportion of this populations have DS, we used validated tools such as the BDI‐II for assessment.^2^, ^16^, ^25^ Using a conservative prevalence estimate of 42.5%, a 95% confidence level (*α* = 0.05), and a margin of error of 5%, the required sample size was estimated using the standard formula: *n* = *Z* ^2^·*p* (1 − *p*)/E^2^, where *Z* = 1.96 for a 95% confidence level, *p* = 0.425 (expected proportion), E = 0.05 (margin of error).^26^ This yielded a minimum requirement of 375 participants. While a 3% error margin would require 1041 participants, we targeted a minimum of 375 to ensure feasibility and adequate precision.

### 2.3 Epilepsy‐related clinical measures

Data collection included seizure type, age at seizure onset, epilepsy diagnosis, and localization. Laterality of the epileptogenic zone was determined using seizure semiology and routine electroencephalography (EEG).

Seizure freedom was defined as no seizures in the 12 months preceding the evaluation. For analytical purposes, participants were stratified according to the ASMs used.

ASMs were categorized into older drugs (Carbamazepine [CBZ], Valproate [VPA], Phenobarbital [PB], Clonazepam [CLZ]) and newer drugs (Topiramate [TPM], Levetiracetam [LEV], Lamotrigine [LTG]). Polytherapy was defined as the concurrent use of two or more ASMs.

We analyzed EEG data collected over the year preceding the study, including a routine 30 min EEG recording. For focal EEG features, morphology, spatial distribution, and location were scored. Morphology was classified as either epileptiform spike, polyspike, or sharp‐wave discharges or delta or theta slowing.^27^ Spatial distribution was scored as single focus, bilateral independent foci, or multifocal graphoelements (two or more independent foci, provided they were not bilateral independent).

### 2.4 Psychiatric assessment

Board‐certified psychiatrists established depression and other psychiatric diagnoses through clinical interviews and medical records review, according to DSM‐5 and ICD‐10 diagnostic criteria. DS and suicidal ideation were assessed as primary outcomes using validated self‐report instruments.

The 21‐item BDI‐II, a self‐administered questionnaire, evaluated DS severity over the past two weeks across cognitive, affective, and somatic domains. It has excellent internal consistency (Cronbach's *α*≈0.90) and robust validity for detecting DS in people with epilepsy.^28^

Additionally, the Georgian version of NDDI‐E, a 6‐item, epilepsy‐specific screen, has good internal consistency (Cronbach's *α* = 0.695), excellent diagnostic accuracy (ROC AUC = 0.975), and strong correlation with BDI scores (Spearman's *ρ* = 0.684). A cutoff score of ≥ 16 yielded 90% sensitivity and 93.9% specificity.^29^, ^30^, ^31^ Suicidal ideations were screened using item 4 of the NDDI‐E (“I'd be better off dead”) with scores > 2 indicating clinically relevant suicidal thoughts.^32^

Other psychiatric and neuropsychological conditions were identified through clinical interviews and medical records review. These were categorized by diagnostic profiles for descriptive purposes only and not analyzed as primary outcomes.

### 2.5 Ethical considerations

The study protocol was approved by the Ethics Committee of the Institute of Neurology and Neuropsychology (#07/IRB‐INN/18). Participants provided verbal informed consent. Written informed consent was waived as participants were adults with full capacity, and participation involved minimal risk.

### 2.6 Data collection and quality control

Adults with epilepsy who expressed interest in participating completed self‐administered questionnaires. Clinical data and investigation results were extracted from medical records and entered into a secure database with standardized quality checks.

### 2.7 Statistical analysis

Statistical analyses were conducted using IBM SPSS Statistics version 28 (IBM Corp., Armonk, NY, USA). Descriptive statistics are presented as frequencies and percentages for categorical variables and medians with interquartile ranges (IQRs) for continuous variables. Normality of distribution was assessed using the Shapiro–Wilk test and visual inspection of histograms and Q–Q plots.

To address the first objective, the prevalence of DS and suicidal ideation was determined using established cutoff scores for the BDI‐II and NDDI‐E. The BDI‐II cutoff score of ≥ 14 was used to indicate mild DS, in accordance with standard interpretation guidelines.^17^ For the NDDI‐E, a cutoff score of ≥ 16 was used to identify clinically significant DS.^29^

To address the second objective, agreement and convergent validity between the BDI‐II and NDDI‐E were evaluated using multiple approaches. Cohen's kappa coefficient was used to assess agreement between categorical classifications based on their respective cutoff scores. Spearman's rank‐order correlation was applied to evaluate the association between total scores, given their non‐normal distribution. Additionally, an intraclass correlation coefficient (ICC) was computed using a two‐way mixed‐effects model with a consistency definition to assess reliability between the instruments when treated as continuous measures.

To address the third objective, clinical and demographic features were compared between participants with and without comorbid DS. Group comparisons were performed as appropriate using the Mann–Whitney U test or Kruskal–Wallis test for non‐normally distributed continuous variables and the Chi‐square test or Fisher's exact test for categorical variables.

*p*‐Values from univariable analyses were adjusted for multiple comparisons using the Benjamini–Hochberg false discovery rate (FDR) procedure. FDR estimations were performed in Python (version 3.12.12) within Google Colab, and FDR‐adjusted *p*‐values (*q*‐values) < 0.05 were considered statistically significant.

Binary logistic regression was used to examine factors associated with DS in people with epilepsy, further addressing the third objective. The dependent variable was DS status (present vs. absent). Independent variables were selected based on theoretical relevance and existing evidence. These included seizure type, frequency, age at index seizure, and epileptogenic zone laterality. Lastly, psychiatric diagnoses were recorded into five larger categories based on psychiatric evaluations using DSM‐5/ICD‐10 criteria. These categories were: no diagnosis, depressive disorders, other mood and anxiety disorders, functional/organic psychiatric disorders, and neurocognitive disorders.

Categorical variables were dummy‐coded using clinically meaningful reference categories and entered simultaneously into the model. The fitness of the model was evaluated via the Omnibus Test of Model Coefficients and pseudo R^2^ values (Cox & Snell, Nagelkerke). Results are reported as odds ratios (ORs) with 95% confidence intervals (CIs). All tests were two‐tailed, with statistical significance defined as *p*‐value < 0.05.

## 3 RESULTS

### 3.1 General characteristics

We included 207 people, with a median age of 30.0 years (interquartile range [IQR]: 24.0–43.0); 56% (*n* = 116) were female. Recruitment details are given in Figure S1.

Among those with focal epilepsy (*n* = 144), 55% (*n* = 79) had temporal lobe epilepsy (TLE), followed by frontal lobe epilepsy (FLE) (19.5%, *n* = 28). The median epilepsy duration and the median age at seizure onset were both 15 years. All were taking ASMs, and 25% (*n* = 51) were on polytherapy. One hundred and twelve (54%) were seizure‐free, and 95 (46%) reported ongoing seizures (Table 1).

**TABLE 1:** General characteristics of participants (*n* = 207).

Variables	*n* (%) or median (IQR)
Age (years)	30.0 (24.0–43.0)
Sex	
Male	91 (44.0%)
Female	116 (56.0%)
Age at epilepsy onset (years)	15.0 (8.0–24.0)
Duration of epilepsy (years)	15.0 (9.0–23.0)
Epilepsy type	
Focal	144 (69.5%)
Generalized	43 (21.0%)
Unclassified	20 (9.5%)
Seizure types	
Focal aware seizures	44 (21.5%)
Focal impaired awareness seizures	113 (54.5%)
Myoclonic seizures	16 (7.5%)
Absences	9 (4.5%)
Absences + Myoclonic seizures	7 (3.5%)
Multi‐type seizures	5 (2.5%)
No seizures	13 (6.5%)
Seizure frequency in the past 12 months	
Non‐convulsive seizures – No	120 (58.0%)
Non‐convulsive seizures – Yes	87 (42.0%)
1–3 per week	20 (9.5%)
1–3 per month	36 (17.5%)
1–3 per year	31 (15.0%)
Tonic–clonic seizures – No	112 (54.0%)
Tonic–clonic seizures – Yes	95 (46.0%)
1–3 per week	9 (4.5%)
1–3 per month	37 (18.0%)
1–3 per year	49 (23.5%)
ASM treatment	
Monotherapy	156 (75.5%)
Polytherapy	51 (24.5%)

*Note*: Percentages have been rounded to the nearest 0.5%. Non‐convulsive seizures include focal aware and focal impaired awareness seizures. Multi‐type seizures refer to participants experiencing more than one seizure type concurrently.

Abbreviations: ASM, antiseizure medication; IQR, interquartile range; MD, median.

### 3.2 Prevalence of depressive symptoms and suicidal ideation

On the BDI‐II, 97 participants (47%) had at least mild DS (score ≥ 14) (Table 2), including 38 (18.5%) mild, 36 (17.5%) moderate, and 23 (11%) severe, while 110 participants (53%) were in the minimal symptom range. On the NDDI‐E, 46 participants (22%) screened positive for DS (score ≥ 16), and 161 (78%) screened negative (Figure S2).

**TABLE 2:** Demographic and Clinical Characteristics Stratified by Comorbid DS (*n* = 207).

Variable	With DS (*n* = 97)	Without DS (*n* = 110)	*p*	*q* (FDR)
Demographics				
Age at evaluation, years, median (IQR)	32.0 (22.5–41.5)	28.5 (20.0–37.0)	0.013	**0.041**
Sex, *n* (%)			0.889	0.907
Male	42 (43.5)	49 (44.5)		
Female	55 (56.5)	61 (55.5)		
Education level, *n* (%)			0.057*	0.089
Secondary	55 (56.5)	45 (41.0)		
Vocational	9 (9.3)	7 (6.4)		
University student	4 (4.0)	9 (8.0)		
University degree	29 (30.0)	49 (44.5)		
Employment status, *n* (%)			0.103*	0.136
Unemployed	62 (64.0)	64 (58.0)		
Employed (professional)	12 (12.5)	28 (25.5)		
Employed (non‐professional)	17 (17.5)	14 (12.5)		
Other (retired/homemaker)	6 (6.0)	4 (3.5)		
Epilepsy characteristics				
Age at seizure onset, years, median (IQR)	15.0 (11.0–27.0)	13.5 (6.0–19.5)	0.009	**0.036**
Duration of epilepsy, years, median (IQR)	14.0 (9.0–22.0)	15.0 (8.0–23.0)	0.554	0.630
Seizure type, *n* (%)			0.255	0.319
Focal epilepsy (localization)	73 (75.3)	71 (64.5)	0.019*	0.053
Temporal lobe	34 (42.5)	45 (53.6)		
Frontal lobe	22 (26.3)	7 (8.3)		
Parietal/occipital	7 (8.8)	7 (8.3)		
Undetermined	16 (20.0)	25 (29.8)		
Generalized epilepsy	17 (17.5)	26 (23.6)		
Unclassified	7 (7.2)	13 (11.8)		

*Note*: Percentages have been rounded to the nearest 0.5%. *p* values were obtained using the Mann–Whitney *U* test for continuous variables and *χ* ^2^ or Fisher's exact test (*) for categorical variables, as appropriate. *q* Values were calculated using the Benjamini–Hochberg false discovery rate (FDR) correction. Bolded *q* values indicate results that remain statistically significant after FDR adjustment.

Abbreviation: DS, depressive symptoms.

Suicidal ideation was assessed using the final item of the NDDI‐E, with 19 participants (9%) screening positive.

### 3.3 Agreement between screening instruments for DS

Cross‐tabulation showed that 21.5% (*n* = 45) of participants screened positive on both instruments, 52.5% (*n* = 109) screened negative on both, and discordant results occurred in 25.0% (*n* = 52) positive only on the BDI‐II and 0.5% (*n* = 1) positive only on the NDDI‐E (Figure S2).

The two instruments demonstrated moderate agreement (Cohen's *κ* = 0.469, *p* < 0.001) and a strong positive correlation between total scores (Spearman's *ρ* = 0.723, *p* < 0.001). ICCs using a two‐way mixed‐effects model indicated moderate reliability: ICC = 0.422 (95% CI: 0.303–0.528) for single measures and ICC = 0.593 (95% CI: 0.465–0.691) for average measures (Figure S2).

### 3.4 Clinical and demographic correlates of depressive symptoms and suicidal ideation

Participants with DS had a higher median age at evaluation (32.0 years; IQR: 22.5–41.5) than those without DS (28.5 years; IQR: 20.0–37.0; Mann–Whitney *U* = 3910.0, *p* = 0.013, *q* = 0.041). Sex distribution was similar between groups (females: 56.5% vs. 55.5%; *p* = 0.889, *q* = 0.907).

The median age at epilepsy onset was slightly higher among participants with DS (15.0 years; IQR: 11.0–27.0) compared to those without DS (13.5 years; IQR: 6.0–19.5; *p* = 0.009, *q* = 0.036). Median duration of epilepsy did not differ between groups (14.0 vs. 15.0 years; *p* = 0.554, *q* = 0.630).

Focal epilepsy was the predominant type in both groups (75.5% vs. 64.5%; *p* = 0.255, *q* = 0.319). Seizure localization showed a trend toward FLE being more frequent among participants with DS and TLE among those without DS, but this did not reach significance after FDR correction (Fisher's exact *p* = 0.019, *q* = 0.053) (Table 2).

### 3.5 Antiseizure medications and DS

The median age at ASM initiation was higher in participants with DS (16.0 years; IQR: 10.5–28.0) than in those without DS (14.0 years; IQR: 5.5–20.0; Mann–Whitney *U* = 4049.5, *p* = 0.009, *q* = 0.036) (Table 3). DS was observed in 60.5% of participants on polytherapy and 42.5% on monotherapy; however, the association with polytherapy did not remain significant after FDR adjustment.

**TABLE 3:** Antiseizure medication (ASM) treatment characteristics stratified by depressive symptoms (DS) (*n* = 207).

Variable	Total N	With DS (*n* = 97)	Without DS (*n* = 110)	*p*	*q* (FDR)
Age at initiation of ASMs, years, median (IQR)	–	16.0 (10.5–28.0)	14.0 (5.5–20.0)	0.009	**0.036**
ASM therapy					
Monotherapy	156	66 (68.0)	90 (82.0)	0.069	0.121
Polytherapy (≥ 2 ASMs)	51	31 (32.0)	20 (18.0)	0.024	0.062
ASM monotherapy class				0.027*	0.074
Older ASMs	98	57 (58.0)	41 (42.0)		
Newer ASMs	50	27 (54.0)	23 (46.0)		
Specific ASM (monotherapy)					
Carbamazepine (CBZ)	68	41 (60.5)	27 (39.5)		
Valproate (VPA)	27	15 (55.5)	12 (44.5)		
Levetiracetam (LEV)	37	20 (54.0)	17 (46.0)		
Lamotrigine (LTG)	11	3 (27.0)	8 (73.0)		
Phenobarbital (PB)	2	0	2 (100)		
Clonazepam (CNZ)	1	0	1 (100)		
Topiramate (TPM)	2	0	2 (100)		

*Note*: Percentages are rounded to the nearest 0.5% and calculated within DS groups. *p* values were obtained using the Mann–Whitney *U* test for continuous variables and *χ* ^2^ or Fisher's exact test (*) for categorical variables, as appropriate. *q* Values were calculated using the Benjamini–Hochberg false discovery rate (FDR) correction. Bolded *q* values indicate results that remain statistically significant after FDR adjustment. Older ASMs refer to first‐generation agents; newer ASMs refer to later‐generation agents.

Abbreviations: CBZ, carbamazepine; CNZ, clonazepam; LEV, levetiracetam; LTG, lamotrigine; PB, phenobarbital; TPM, topiramate; VPA, valproate.

CBZ was the most common ASM. 77.5% of those on CBZ monotherapy had DS, compared with 24.0% on VPA monotherapy. LEV use was higher in those with DS (32%). Group‐level differences across ASM types were not statistically significant after FDR correction.

High‐dose ASM regimens were more prevalent in the DS group (31%) than in the non‐DS group (14.5%), while low‐dose regimens were more common in the non‐DS group (6% vs. 14.6%). A significant linear trend across dosage levels (Linear‐by‐Linear Association, *p* = 0.010, *q* = 0.036) was observed, which remained significant after FDR correction.

### 3.6 Psychiatric and neuropsychological problems

The distribution of psychiatric and cognitive comorbidities differed significantly between participants with and without DS (Table 4). Depression, with or without comorbid anxiety, occurred exclusively in the DS group (39 participants, 40%). Mood and anxiety disorders were more prevalent in the DS group (43 participants, 44.5%) than in those without DS (26 participants), an association that remained significant after FDR adjustment.

**TABLE 4:** Psychiatric and cognitive diagnoses in people with epilepsy with and without DS (*n* = 207).

Characteristics	With DS (*n* = 97)	Without DS (*n* = 110)	*p*	*q* (FDR)
Any psychiatric/neuropsychological diagnosis			< 0.001	**< 0.001**
Depression with or without anxiety disorders	39 (40.0)			
Mood or anxiety disorders (without depression)	43 (44.5)	26 (23.5)		
Dissociative and sleep disorders	10 (10.5)	7 (6.5)		
Non‐progressive cognitive impairment/organic psychiatric conditions	5 (5.0)	11 (10.0)		
Suicidal thoughts (yes)	19 (19.5)			
Psychiatric medication use (yes)	27 (28.0)	24 (22.0)	0.010	0.036

*Note*: Percentages are rounded to the nearest 0.5% and calculated within DS groups. *p*‐values were derived using *χ* ^2^ or Fisher's exact test, as appropriate. *q* values were estimated using the Benjamini–Hochberg false discovery rate (FDR) procedure. Bolded *q* values indicate results that remain statistically significant after FDR correction.

Dissociative and sleep disorders occurred in 10 participants (10.5%) with DS and in 7 (6.5%) without, which was not significant after FDR correction. Non‐progressive cognitive impairment and other organic psychiatric conditions were more common in those without DS (11 participants, 10%) than in those with DS (5 participants, 5%), although the difference was not significant.

Psychiatric medication use was higher among participants with DS (27 participants, 28%) than those without DS (24 participants), remaining significant after FDR adjustment.

These observations were derived from psychiatrist‐conducted clinical interviews, medical record reviews, and standardized psychiatric screening instruments rather than from formal neuropsychological testing. Most participants had psychiatric conditions that had not been formally assessed or treated before the study.

Participants with suicidal ideation had a higher prevalence of psychiatric comorbidities, particularly DS and anxiety disorders (89.5% vs. 48%). While most epilepsy‐related variables were similar between groups, those with suicidal ideation had a shorter median duration of epilepsy. A higher proportion of females was identified in those with suicidal ideation (79% vs. 53.5%); however, this difference was not significant after FDR correction (Table 5).

**TABLE 5:** Suicidal ideation characteristics in people with epilepsy (*n* = 207).

Characteristics	Suicidal ideation positive (*n* = 19)	Suicidal ideation negative (*n* = 188)	*p*	*q* (FDR)
Age, median (IQR), years	29.0 (25.0–42.0)	30.0 (24.0–43.0)	0.648	0.704
Sex (female), *n* (%)	15 (79.0)	101 (53.5)	0.050	0.083
BDI‐II total score, median (IQR)	25.0 (21.0–38.5)	12.0 (7.0–18.5)	< 0.001	**0.008**
Any psychiatric comorbidity (yes), *n* (%)	17 (89.5)	90 (48.0)	< 0.001	**0.008**
Major depression diagnosis, *n* (%)	13 (68.5)	12 (6.5)	< 0.001	**0.008**
Psychiatric medication use (yes), *n* (%)	8 (42.0)	86 (47.0)	0.907	0.907

*Note*: Analyses are exploratory. *p* Values were derived using the Mann–Whitney *U* test for continuous variables and Fisher's exact test for categorical variables. *q* Values were calculated using the Benjamini–Hochberg false discovery rate (FDR) procedure. Bolded *q* values indicate results that remain statistically significant after FDR correction.

Abbreviation: BDI‐II, Beck Depression Inventory‐II.

### 3.7 Multivariable analysis

A multivariable logistic regression was performed to identify factors independently associated with DS among people with epilepsy. The overall model was statistically significant (Omnibus Test of Model Coefficients: *χ* ^2^[10] = 30.66, *p* = 0.001), explaining 14.0%–18.6% of the variance in DS status. Model convergence was achieved after four iterations with stable parameters (< 0.001 change in −2 log‐likelihood).

Age at first seizure was positively associated with DS, with each 1‐year increase corresponding to a 5% increase in odds (OR = 1.05, 95% CI: 1.02–1.08, p = 0.001). Seizure laterality was also significant (*p* = 0.044); participants with a right‐sided seizure focus had lower odds of DS compared to the reference group (OR = 0.24, 95% CI: 0.09–0.61, *p* = 0.007). Seizure‐freedom was associated with reduced odds of DS (OR = 0.39, 95% CI: 0.18–0.83, *p* = 0.014). Seizure type was not significantly associated with DS (*p* = 0.250). A prior psychiatric diagnosis was linked to higher odds of DS (OR = 1.38, 95% CI: 0.96–1.99), though this did not reach statistical significance (*p* = 0.076) (Table 6).

**TABLE 6:** Factors independently associated with DS (*n* = 207).

Variable	OR (95% CI)	*p*
Age at index seizure (years)	1.05 (1.02–1.08)	0.001
Seizure type (overall)	–	0.250
Focal aware	0.95 (0.35–2.59)	0.947
Focal impaired awareness	1.15 (0.37–3.56)	0.818
Generalized	0.51 (0.15–1.75)	0.359
Unclassified	0.51 (0.12–2.17)	0.324
Laterality on EEG (overall)	–	0.044
Right‐sided	0.24 (0.09–0.62)	0.007
Left‐sided	0.57 (0.23–1.41)	0.245
Bilateral/Uncertain	0.96 (0.41–2.26)	0.934
Ongoing seizures vs seizure‐free	0.39 (0.18–0.83)	0.014
Psychiatric diagnosis	1.38 (0.95–2.01)	0.076

*Note*: Seizure type and laterality are categorical variables with overall *p*‐values reported; subcategories are compared to reference groups. Psychiatric diagnosis is a binary variable indicating the presence of any psychiatric comorbidity.

## 4 DISCUSSION

Our findings show that DS and psychiatric comorbidities are common among people with epilepsy and frequently underrecognized. Prevalence aligns with international reports while providing novel data from Georgia. Suicidal ideation was identified in a proportion of participants, underscoring the clinical importance of routine mental health assessment in epilepsy care. Given the well‐established association between epilepsy and suicide risk, early identification and monitoring of at‐risk individuals are critical.

Screening tools demonstrated important clinical implications. The NDDI‐E appears to capture core depressive features while avoiding somatic symptoms that may overlap with seizure manifestations.^18^, ^33^, ^34^ Moderate agreement between the NDDI‐E and BDI‐II highlights both strengths and limitations. Despite a strong correlation, discordant cases were frequent; psychiatric evaluation confirmed that many individuals identified only by the BDI‐II had clinically meaningful DS. This suggests that reliance on the NDDI‐E alone may underestimate clinically relevant symptoms, whereas broader tools may increase sensitivity at the expense of specificity. Positive screening results should prompt further evaluation by qualified mental health professionals. A structured pathway, including an initial brief screening with the NDDI‐E followed by a comprehensive assessment using the BDI‐II or psychiatric evaluation in borderline or discordant cases, may optimize detection. It should also minimize false positives and negatives.

Several demographic factors were associated with DS. Participants with DS were generally older and had later epilepsy onset, with age at onset remaining independently associated after adjustment. Even modest differences in adolescent and early adult onset may influence vulnerability to DS, consistent with prior studies.^4^, ^19^, ^34^ Unlike general population trends, no sex differences in DS were observed, suggesting that epilepsy‐specific neurobiological or psychosocial factors may modify typical sex‐related risk patterns.^35^, ^36^, ^37^ Participants without DS tended to have higher levels of education and employment status, although these differences were not statistically significant after multiple‐comparison adjustment. Nonetheless, lower educational attainment and unemployment remain important determinants of poorer psychosocial outcomes and DS burden.^38^

Differences in the distribution of epileptic foci emerged in relation to DS status. FLE was more frequently observed among participants with DS, whereas TLE predominated among those without. These findings align with some prior reports highlighting the role of frontal networks in mood regulation and executive function.^6^, ^8^, ^39^, ^40^, ^41^ However, an extensive body of work also supports higher rates of depression in people with TLE, particularly where limbic structures such as the hippocampus and amygdala are involved. For example, meta‐analytic evidence indicates that hippocampal sclerosis (a hallmark of mesial TLE) is associated with an increased likelihood of depression.^42^, ^43^, ^44^ EEG laterality also appeared to be relevant: individuals with right‐sided seizure foci had lower odds of DS, a pattern echoed in select neuroimaging and neuropsychological investigations.^45^

Treatment patterns and clinical management were also related to DS. Participants with DS were more frequently prescribed polytherapy and received higher ASM doses, although some associations lost significance after multiple comparison adjustment.

Participants with DS had more frequent seizures, functional or organic psychiatric conditions, and mood or anxiety disorders, in line with prior reports.^2^, ^3^, ^12^, ^46^, ^47^ Suicidal ideation occurred among people with DS, with half untreated, emphasizing the need for integrated mental health services within epilepsy care in Georgia.

Multivariable analysis suggests that the risk of developing DS and psychiatric comorbidities increases with epilepsy progression and is significantly higher among those with uncontrolled seizures.

Overall, these findings highlight the need for periodic mental health screening in people with epilepsy, which provides opportunities for early prevention, timely diagnosis, and adequate management of psychiatric disorders. By providing Georgia‐specific data on DS and suicidal ideation, this study offers an empirical basis for informing national policies, guiding integration of mental health services into epilepsy care, planning clinical services, and optimizing resource allocation within the country's healthcare framework.

### 4.1 Strengths and limitations

Prospective design and consecutive sampling enhanced representativeness and reduced selection bias, while standardized diagnostic criteria and validated screening tools supported reliable psychiatric assessment. Detailed clinical data enabled comprehensive characterization of epilepsy and psychiatric comorbidity, and adjustment for multiple comparisons using the Benjamini–Hochberg FDR procedure strengthened the reliability of reported associations.

The sample size was smaller than planned due to high exclusion and refusal rates, raising concerns about representativeness. The burden of participation, including multiple questionnaires and psychiatric evaluation, may have reduced participation, particularly among individuals with greater psychosocial burden, potentially influencing prevalence estimates and sample composition.

This may have introduced selection bias affecting screening outcomes and their concordance with psychiatric evaluation, as participants completing multiple assessments may differ in symptom awareness or reporting behavior.

Additional limitations include single‐centre recruitment, which limits generalisability; reliance on self‐report instruments, which are subject to reporting bias; and the absence of structured psychiatric interviews and ASM‐level data. Some subgroup analyses involved small cell sizes, which may affect estimate stability, and the cross‐sectional design precludes conclusions about causal or temporal relationships.

Despite these limitations, the overall design and use of validated tools alongside psychiatric assessment support the internal validity of the findings. Future studies with larger, more representative samples and reduced participation burden are warranted.

## 5 CONCLUSION

Mental health issues are common but underrecognized in Georgian adults with epilepsy. To improve care, clinicians should adopt a stepped screening approach using specific tools, such as the NDDI‐E. Routine monitoring is essential, particularly for those with severe epilepsy. Future work should focus on developing culturally appropriate, resource‐sensitive screening and treatment strategies.

## AUTHOR CONTRIBUTIONS

K. S., S. K., T. S., and J. W. S. conceived and designed the study. K. S., M. A., and E. B. managed data and performed analyses. K. S., E. B., M. A., T. S., and S. K. drafted the manuscript. K. S., E. B., S. K., and J. W. S. critically revised the text. J. W. S. provided overall supervision and conceptual guidance. All authors read and approved the final version of the manuscript.

## FUNDING INFORMATION

This work was supported by the Young Scientists' Research Grant of the Caucasus International University, Tbilisi, Georgia [CIU‐SG‐127‐18].

## CONFLICT OF INTEREST STATEMENT

The authors declare no conflict of interest in relation to this work.

## ETHICAL STATEMENT

We confirm that we have read the Journal's position on issues involved in ethical publication and affirm that this report is consistent with those guidelines.

## SOCIAL MEDIA AND ARTICLE PROMOTION

Depressive symptoms and suicidal ideation are highly prevalent among adults with epilepsy in Georgia, highlighting critical unmet mental health needs.

#Epilepsy #Depression #SuicidalIdeation #MentalHealth #Neurology #EpilepsyResearch.

## Supporting information


**FIGURE S1.** Recruitment flowchart of study participants. Abbreviations: INN, Institute of Neurology and Neuropsychology; ASM, Antiseizure medication.

**FIGURE S2.** Depression screening outcomes using BDI‐II and NDDI‐E Abbreviations: BDI‐II – Beck Depression Inventory‐II; NDDI‐E: Neurological Disorders Depression Inventory for Epilepsy. ‐ Percentages are calculated using the total sample size (*n* = 207) and rounded to the nearest 0.5%.

**FIGURE S3.** Distribution of the Depression Symptoms by BDI‐II and NDDI‐E Abbreviations: BDI‐II – Beck Depression Inventory‐II; NDDI‐E: Neurological Disorders Depression Inventory for Epilepsy ‐ Percentages are calculated using the total sample size (*n* = 207) and rounded to the nearest 0.5%.

## Data Availability

The data that support the findings of this study are available on request from the corresponding author. The data are not publicly available due to privacy or ethical restrictions.
