# Supplementary figures and images for: Occurrence and clinical correlates of depressive symptoms in adults with epilepsy: A study in Georgia
Source: Epilepsia Open. 2026 Jun 8;11(4):1290–300. doi: 10.1002/epi4.70291 (PMC13394209; doi:10.1002/epi4.70291)

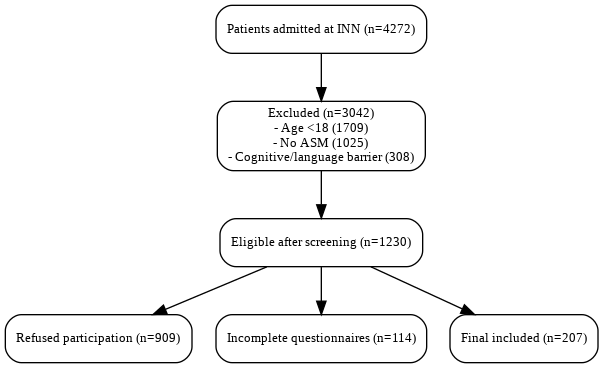

Supplement: Supplementary file 1 — FIGURE S1. Recruitment flowchart of study participants. Abbreviations: INN, Institute of Neurology and Neuropsychology; ASM, Antiseizure medication. [file EPI4-11-1290-s001.tif]

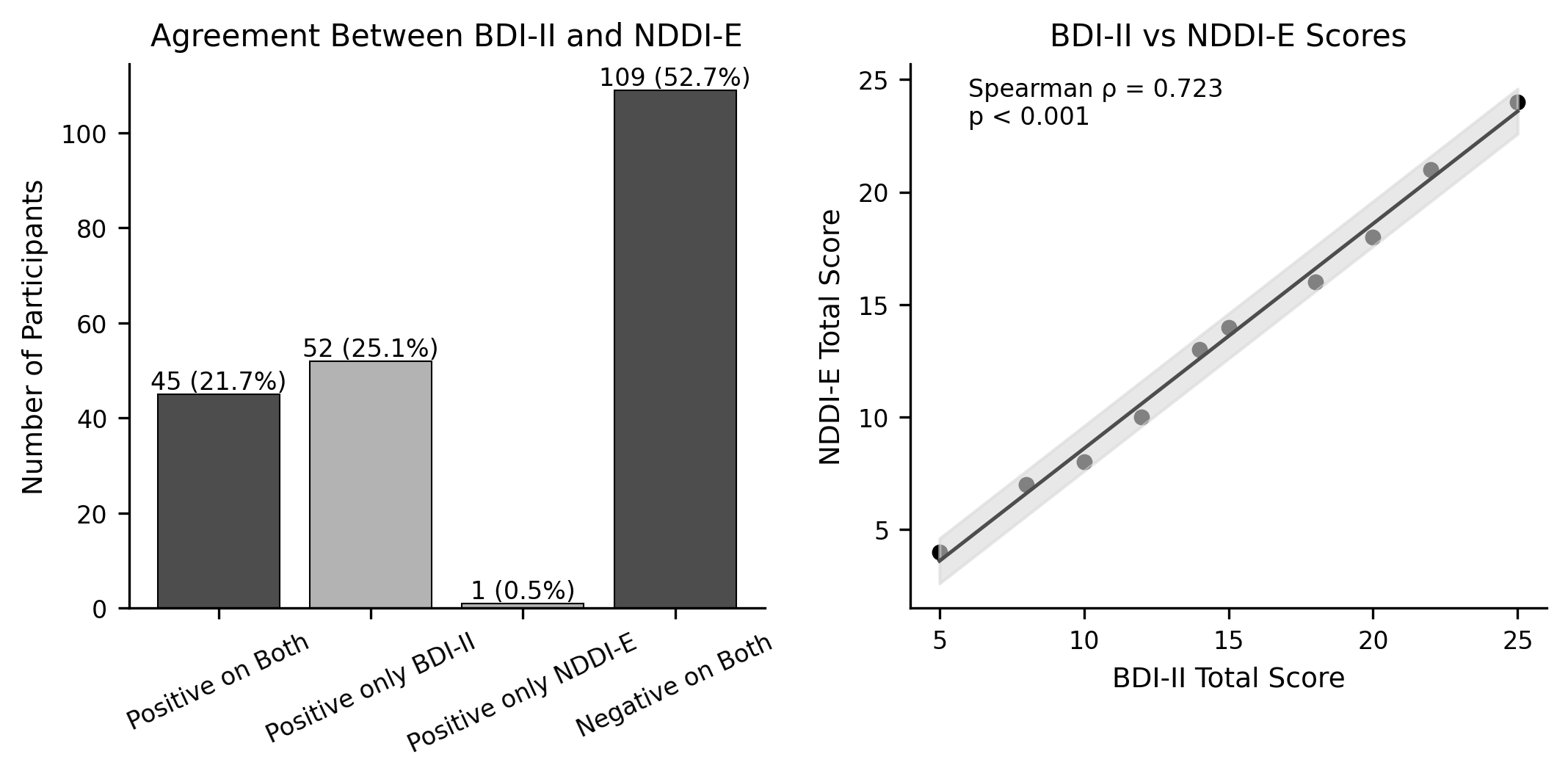

Supplement: Supplementary file 2 — FIGURE S2. Depression screening outcomes using BDI‐II and NDDI‐E Abbreviations: BDI‐II – Beck Depression Inventory‐II; NDDI‐E: Neurological Disorders Depression Inventory for Epilepsy. ‐ Percentages are calculated using the total sample size (n = 207) and rounded to the nearest 0.5%. [file EPI4-11-1290-s002.tif]

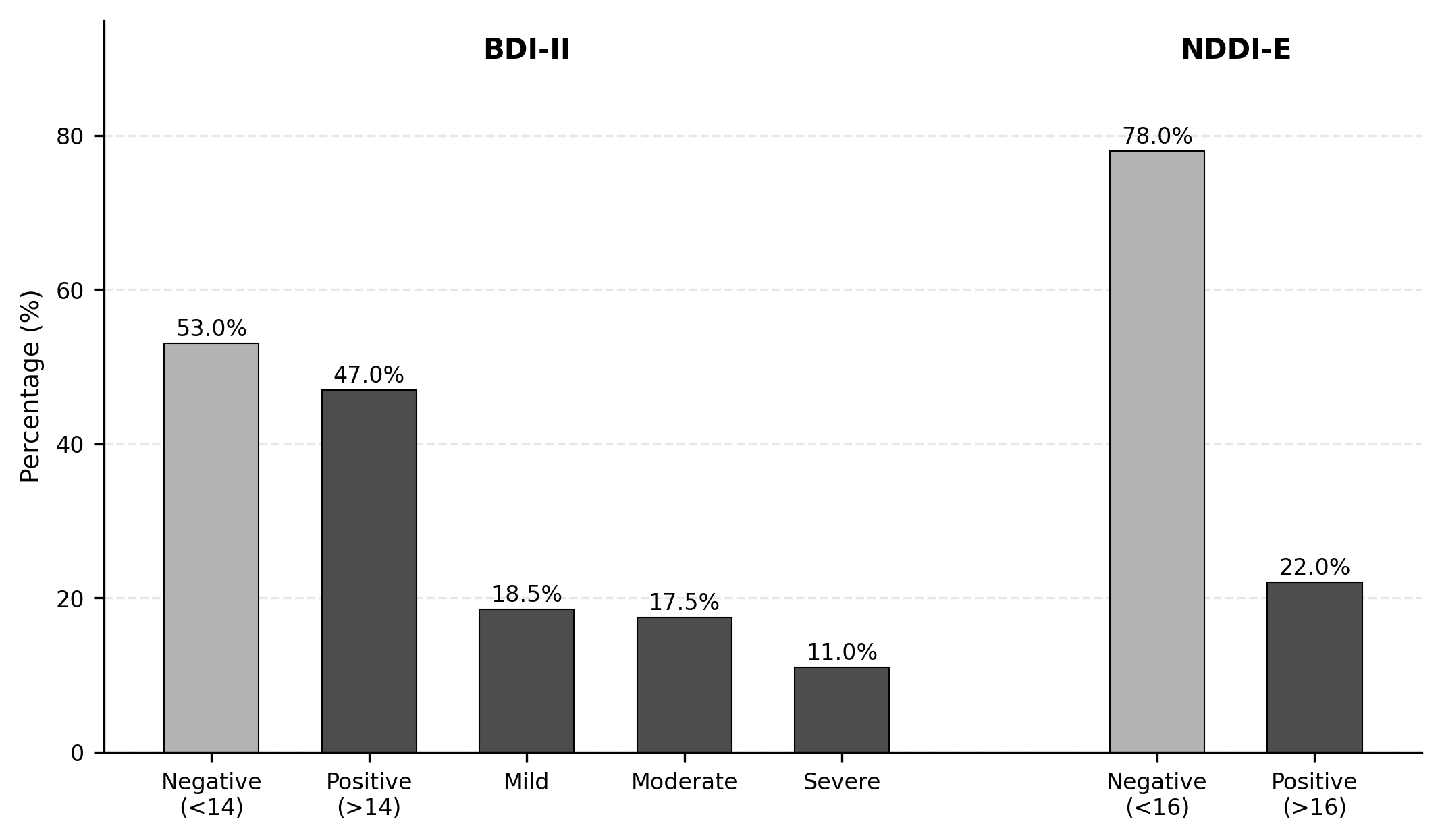

Supplement: Supplementary file 3 — FIGURE S3. Distribution of the Depression Symptoms by BDI‐II and NDDI‐E Abbreviations: BDI‐II – Beck Depression Inventory‐II; NDDI‐E: Neurological Disorders Depression Inventory for Epilepsy ‐ Percentages are calculated using the total sample size (n = 207) and rounded to the nearest 0.5%. [file EPI4-11-1290-s003.tif]
